# Metallothionein-1 as a biomarker of altered redox metabolism in hepatocellular carcinoma cells exposed to sorafenib

**DOI:** 10.1186/s12943-016-0526-2

**Published:** 2016-05-16

**Authors:** Aline Houessinon, Catherine François, Chloé Sauzay, Christophe Louandre, Gaelle Mongelard, Corinne Godin, Sandra Bodeau, Shinichiro Takahashi, Zuzana Saidak, Laurent Gutierrez, Jean-Marc Régimbeau, Nathalie Barget, Jean-Claude Barbare, Nathalie Ganne, Bruno Chauffert, Romain Coriat, Antoine Galmiche

**Affiliations:** Laboratoire de Biochimie, Centre de Biologie Humaine (CBH), CHU Amiens Sud, Avenue Laennec, 80054 Amiens, Cedex France; EA4666, Université de Picardie Jules Verne (UPJV), Amiens, France; EA4294, Université de Picardie Jules Verne (UPJV), Amiens, France; Centre de Ressources en Biologie Moléculaire, UPJV, Amiens, France; Service de Pharmacologie, CHU Sud, Amiens, France; Department of clinical laboratory, Tohoku Medical and Pharmaceutical University Hospital, Sendai, Japan; Service de Chirurgie Viscérale, CHU Sud, Amiens, France; Centre de Ressources Biologiques, Hôpitaux Universitaires Paris Seine-Saint-Denis, APHP, Bondy, France; Service d’Hépatologie, Pôle d’Activités Cancérologiques Spécialisées, APHP, Hôpitaux Universitaires Paris Seine-Saint-Denis, Site Jean Verdier, Bondy, France; Université Paris 13, Sorbonne Paris Cité, UFR SMBH, Bobigny, France; Inserm UMR-1162, Génomique Fonctionnelle des Tumeurs solides, Paris, France; Service de Gastroentérologie et d’Endoscopie, Hôpital Cochin, APHP, Paris, France; Université Paris Descartes, Sorbonne Paris Cité, UFR de médecine, Paris, France

**Keywords:** Metallothionein-1, Sorafenib, Hepatocellular carcinoma, Redox metabolism, Ferroptosis, Biomarker

## Abstract

**Background:**

Sorafenib, a kinase inhibitor active against various solid tumours, induces oxidative stress and ferroptosis, a new form of oxidative necrosis, in some cancer cells. Clinically-applicable biomarkers that reflect the impact of sorafenib on the redox metabolism of cancer cells are lacking.

**Methods:**

We used gene expression microarrays, real-time PCR, immunoblot, protein-specific ELISA, and gene reporter constructs encoding the enzyme luciferase to study the response of a panel of cancer cells to sorafenib. Tumour explants prepared from surgical hepatocellular carcinoma (HCC) samples and serum samples obtained from HCC patients receiving sorafenib were also used.

**Results:**

We observed that genes of the metallothionein-1 (MT1) family are induced in the HCC cell line Huh7 exposed to sorafenib. Sorafenib increased the expression of *MT1G* mRNA in a panel of human cancer cells, an effect that was not observed with eight other clinically-approved kinase inhibitors. We identified the minimal region of the *MT1G* promoter that confers inducibility by sorafenib to a 133 base pair region containing an Anti-oxidant Response Element (ARE) and showed the essential role of the transcription factor NRF2 (Nuclear factor erythroid 2-Related Factor 2). We examined the clinical relevance of our findings by analysing the regulation of *MT1G* in five tumour explants prepared from surgical HCC samples. Finally, we showed that the protein levels of MT1 increase in the serum of some HCC patients receiving sorafenib, and found an association with reduced overall survival.

**Conclusion:**

These findings indicate that MT1 constitute a biomarker adapted for exploring the impact of sorafenib on the redox metabolism of cancer cells.

**Electronic supplementary material:**

The online version of this article (doi:10.1186/s12943-016-0526-2) contains supplementary material, which is available to authorized users.

## Background

Sorafenib, a multikinase inhibitor with a relatively broad spectrum, is a medically-approved treatment efficacious against advanced hepatocellular carcinoma (HCC) and other types of solid tumours [1]. In addition to its inhibitory effect on RAF and Vascular Endothelial Growth Factor Receptor (VEGFR) kinases [2], it is increasingly clear that sorafenib possesses other biochemical activities that might contribute to its anti-oncogenic efficacy [3]. Identification of specific biomarkers that could be used for the analysis of the contribution of each mode of action is needed in order to understand how sorafenib exerts its antioncogenic activity, and to allow for eventual repurposing and personalization of the prescription of this drug in clinics.

Recently, we and others reported that sorafenib is an inducer of oxidative stress [4–7], eventually leading to the occurrence of a new form of regulated necrosis, called ferroptosis, in various human cancer cell lines established from HCC and other solid tumours [5–9]. Ferroptosis is a form of necrosis characterized by membrane lipid peroxidation [10, 11]. Interestingly, the ability of sorafenib to induce ferroptosis is not shared by other, clinically-approved kinase inhibitors, and is therefore probably unrelated to its inhibitory action on its essential target kinases [8]. The ability of sorafenib to induce ferroptosis might be partially explained by its ability to interfere with the function of the Xc(-) membrane transporter [6], a membrane transporter for the amino-acid cystine, a rate-limiting precursor for the synthesis of Glutathione (GSH) [6]. In addition to depleting the intracellular GSH levels, sorafenib increases the mitochondrial production of reactive oxygen species (ROS) in HCC cells [4, 9]. Interestingly, Coriat et al. found high levels of advanced oxidation products of proteins (AOPP), a marker of oxidative stress, in the serum of HCC patients treated with sorafenib [4]. While the findings of Coriat et al. suggest that this new mode of action of sorafenib on the redox metabolism of cancer cells might be relevant to the clinical setting, AOPP do not constitute a specific marker and their measurement is not easily standardized [12, 13]. The identification of new biomarkers that would directly reflect the activity of sorafenib on the redox metabolism of cancer cells is therefore awaited.

Metallothioneins (MT) are a family of small intracellular proteins that are ubiquitously expressed by eukaryotic cells and are characterized by a common structure and a high content of the amino-acid Cysteine [14, 15]. In humans, MTs are encoded by 17 genes defining four groups (MT1-MT4). The first group, called MT1, is the largest and consists of 13 members. Members of this group are important regulators of the homeostasis of zinc and copper ions, and play a role in the defence mechanisms of eukaryotic cells against heavy metals such as Cadmium [15]. MT1 also represent an important cellular defence against oxidative stress in various cell types [15]. Multiple alterations of the expression of individual isoforms of MT1 have been reported in solid tumours, some of which may be associated with the patient’s prognosis [16]. Individual isoforms of MT1 have also been reported to modulate the response of tumour cells to chemotherapeutic agents [17–19]. There is however little literature regarding the regulation and the possible role that MT1 might play in cancer cells exposed to targeted therapies, such as sorafenib.

## Results

### Sorafenib increases the expression of MT1 genes in cancer cells

**Fig. 1:**
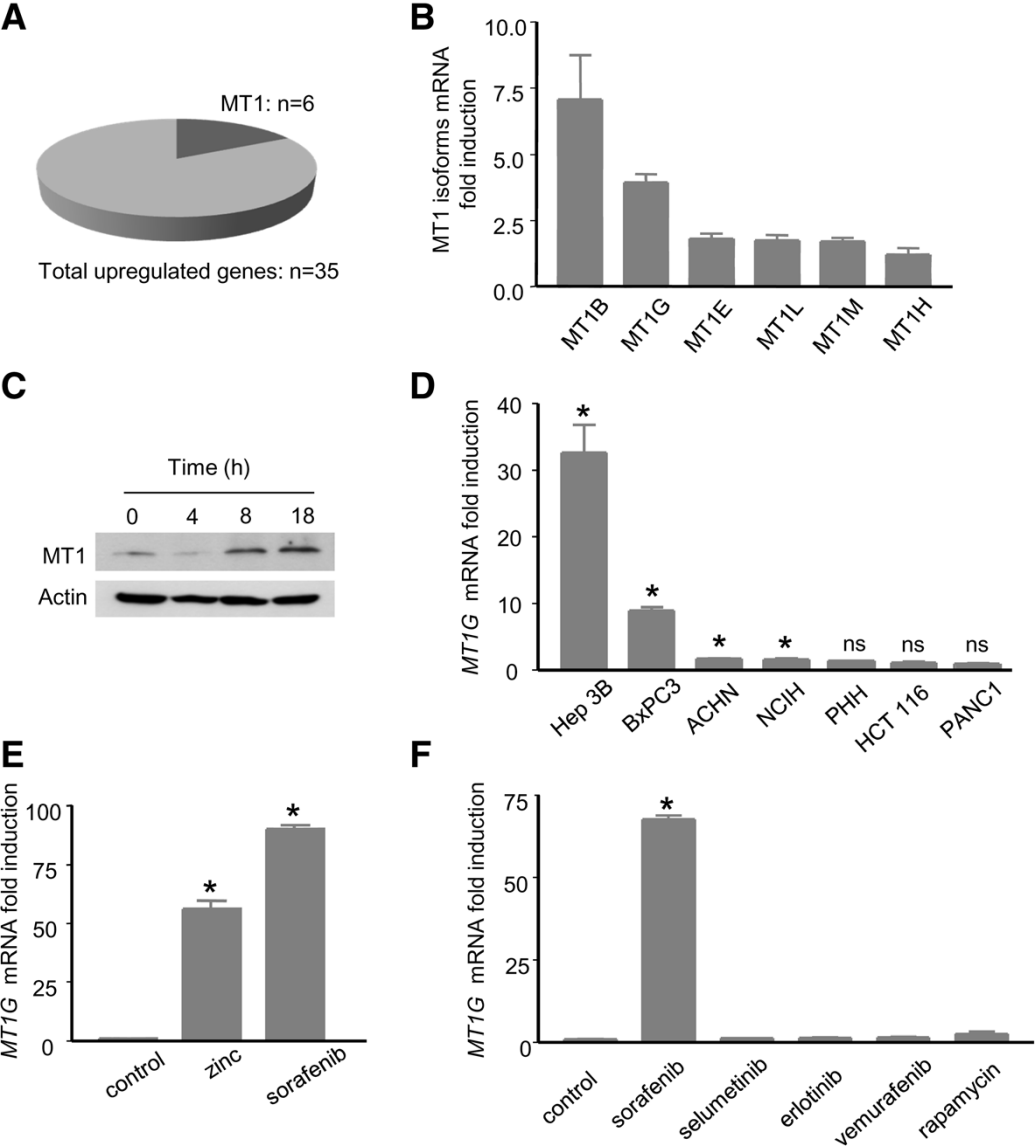
*Sorafenib increases the expression levels of multiple MT1 genes in cancer cells*. **a** *MT1* genes represent a significant fraction (6/35) of the genes found to be overexpressed by more than three-fold in Huh7 cells exposed to sorafenib (10 μM) for 9 h. **b** QPCR analysis of the mRNA encoding the main isoforms of MT1 in Huh7 cells exposed to sorafenib (10 μM) for 9 h. **c** Immunoblot analysis of the expression of the MT1 protein. Protein extracts were prepared from Huh7 cells treated for 4 to 18 h with 10 μM sorafenib. **d** *MT1G* mRNA levels were measured by QPCR in a panel of cancer cell lines and in primary human hepatocytes (PHH) exposed to sorafenib 10 μM for 9 h. *: *p* < 0.05 compared to control, n.s.: non-significant compared to control. **e**: *MT1G* mRNA levels analysis in Huh7 cells exposed to ZnCl_2_ 100 μM and sorafenib (10 μM) for 18 h. **f** Analysis of *MT1G* mRNA levels by QPCR in Huh7 cell lines exposed to sorafenib or selected kinase inhibitors applied at pharmacological concentrations (selumetinib 1 μM, erlotinib 1 μM, vemurafenib 10 μM and rapamycin 1 μM) for 18 h. *: *p* < 0.05 compared to control

In order to explore the response of HCC cells to sorafenib, we examined the transcriptome of Huh7 cells exposed to sorafenib (10 μM, 9 h) with genome-wide microarrays. Interestingly, of the 35 genes that were found to be overexpressed by more than 3-fold upon sorafenib exposure, six were members of the MT1 family (Fig. 1a, see Additional file 1: Table S1). Quantitative PCR confirmed the induction of these six genes (*MT1B, MT1G, MT1E, MT1L, MT1M and MT1H*), confirming the results of the microarray (Fig. 1b). Immunoblot analysis also confirmed the induction of MT1 at the protein level (Fig. 1c). These findings prompted us to examine the regulation of MT1 in cancer cells exposed to sorafenib. The analysis of the threshold cycle (Ct) suggested that the *MT1G* gene was one of the most abundant isoforms of the MT1 family at the mRNA level in Huh7 cells. Since *MT1G* was also one of the isoforms whose expression was most strongly induced by sorafenib, we centred our analysis on the regulation of this isoform. We examined the effect produced by sorafenib in a panel of cancer cell lines originating from different types of human tumours (Fig. 1d). Sorafenib was found to induce a robust increase in the levels of the mRNA encoding MT1G in the human cell lines Hep3B and BxPC3 established from HCC and Pancreatic adenocarcinoma, respectively (Fig. 1d). In the other cells, the induction was either statistically-significant but of low amplitude (ACHN, NCIH) or absent (HCT116, PANC1, primary human hepatocytes) (Fig. 1d). In each cell line, except for Huh7 cells, we observed that sorafenib had little effect on cell viability. In Huh7 cells, overnight exposure to 10 μM sorafenib reduced cell viability by 15 %, shown by trypan blue exclusion. The loss of viability never exceeded 5 % in the other cell lines (data not shown). The effect of sorafenib on *MT1G* mRNA was therefore unrelated to its effect on cell viability. In Huh7 cells, the induction of the *MT1G* mRNA was robust, and the effect produced by sorafenib was even superior to that of ZnCl_2_, the inducer of reference of MT1, in terms of potency for the induction of *MT1G* (Fig. 1e). A similar pattern of induction was found with the *MT1B* mRNA (see Additional file 1: Figure S1). We wondered whether other chemical inhibitors of kinases, applied at pharmacological concentrations, would induce an increase in the expression levels of *MT1G* mRNA (Fig 1f). The following chemical inhibitors were applied to Huh7 cells: erlotinib (1 μM), directed against the Epidermal Growth Factor Receptor (EGFR), vemurafenib (10 μM), directed against the BRAF kinase, selumetinib (1 μM), directed against the kinases MEK1/2, and rapamycin (1 μM), directed against the kinase mammalian Target of Rapamycin (mTOR). None of these inhibitors increased the expression of *MT1G* in Huh7 cells (Fig. 1f).

### Oxidative stress and cysteine metabolism play an important role in the induction of the MT1G gene by sorafenib

**Fig. 2:**
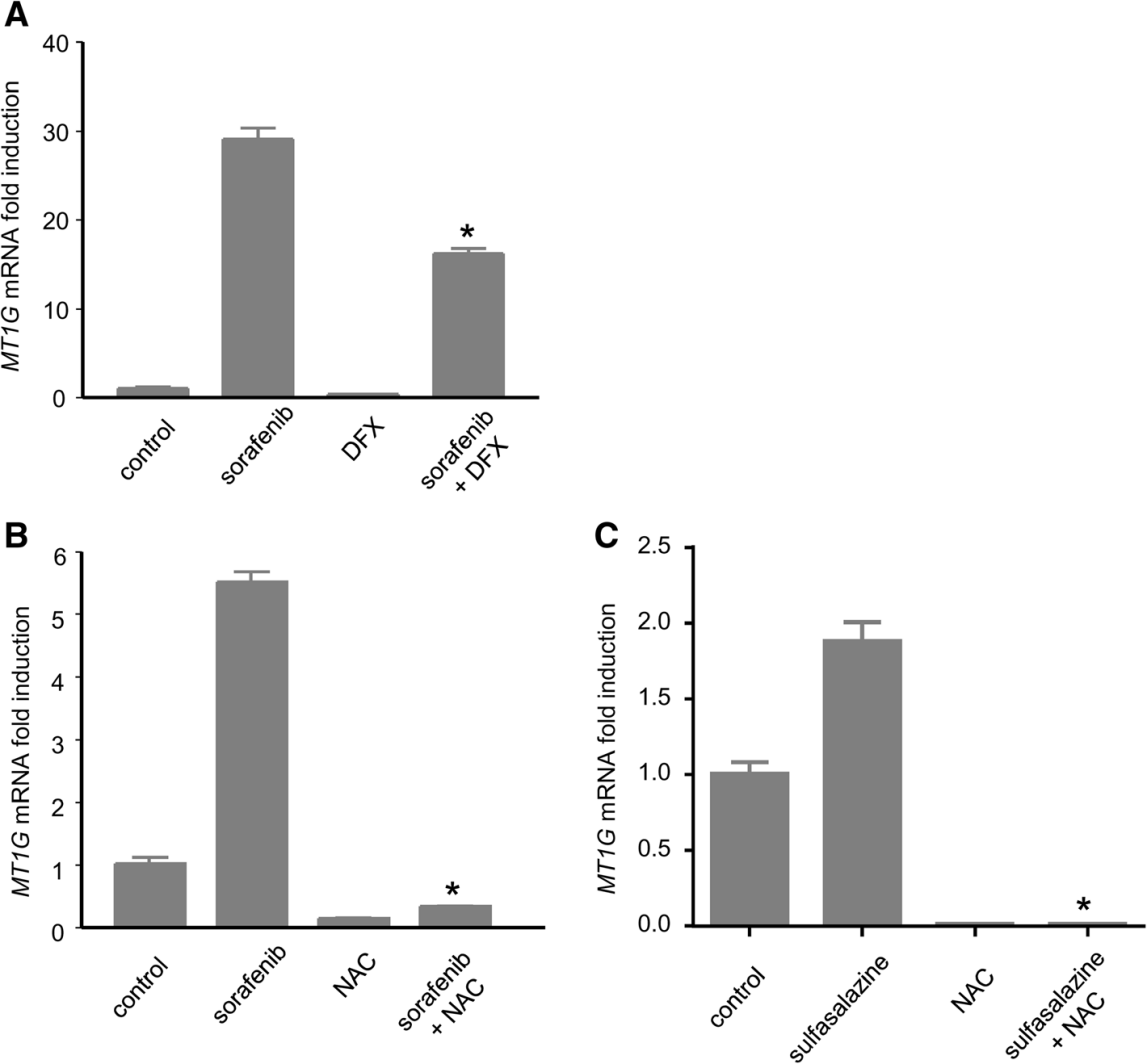
*Pharmacological antioxidants prevent the induction of MT1G induced by sorafenib*. **a**: *MT1G* mRNA induction in Huh7 cells treated with sorafenib 10 μM and DFX 100 μM (preincubated 1 h) for 18 h. **b** Induction of *MT1G* mRNA in Huh7 cells exposed for 18 h to sorafenib 10 μM and/or NAC 10 mM (preincubated 1 h). *: *p* < 0.05 compared to sorafenib alone. **c** Induction of MT1G mRNA in Huh7 cells exposed to 300 μM sulfasalazine and/or NAC 10 mM (preincubated 1 h) for 18 h. *: *p* < 0.05 compared to sulfasalazine alone

In order to explore the possibility that oxidative stress might play a role in the overexpression of MT1 induced by sorafenib, Huh7 cells were treated with deferoxamine (DFX), an iron chelator reported to protect cancer cells from oxidative stress and ferroptosis by preventing the formation of potent oxidants [5, 10] before being exposed to sorafenib (Fig. 2a). We found that DFX reduced the levels of *MT1G* mRNA in Huh7 cells exposed to sorafenib (10 μM, 18 h) (Fig. 2a). In order to directly address the possibility that sorafenib might regulate the levels of *MT1G* via its ability to interfere with the transport of the amino-acid cystine [6], we preincubated Huh7 cells with N-Acetyl-Cysteine (NAC), a chemical precursor of cysteine and a potent antioxidant, before treatment with sorafenib (Fig. 2b). We found that NAC radically prevented the increase in *MT1G* expression induced by sorafenib (Fig. 2b). Comparable results were obtained with *MT1B* (see Additional file 1: Figure S2). In order to further examine the impact of cystine transport inhibition on the regulation of *MT1G*, we applied sulfasalazine, a chemical inhibitor of the membrane transporter Xc(-) structurally-unrelated to sorafenib [20] to Huh7 cells. Sulfasalazine increased *MT1G* mRNA levels at a pharmacologically-active concentration of 300 μM [20], an effect that was blocked by NAC (Fig. 2c). These findings pointed to oxidative stress and cysteine metabolism as playing a possible role in the regulation of *MT1* gene expression in cancer cells exposed to sorafenib.

### Transcriptional regulation of the MT1G promoter by the transcription factor NRF2 (Nuclear factor erythroid 2-Related Factor 2)

**Fig. 3:**
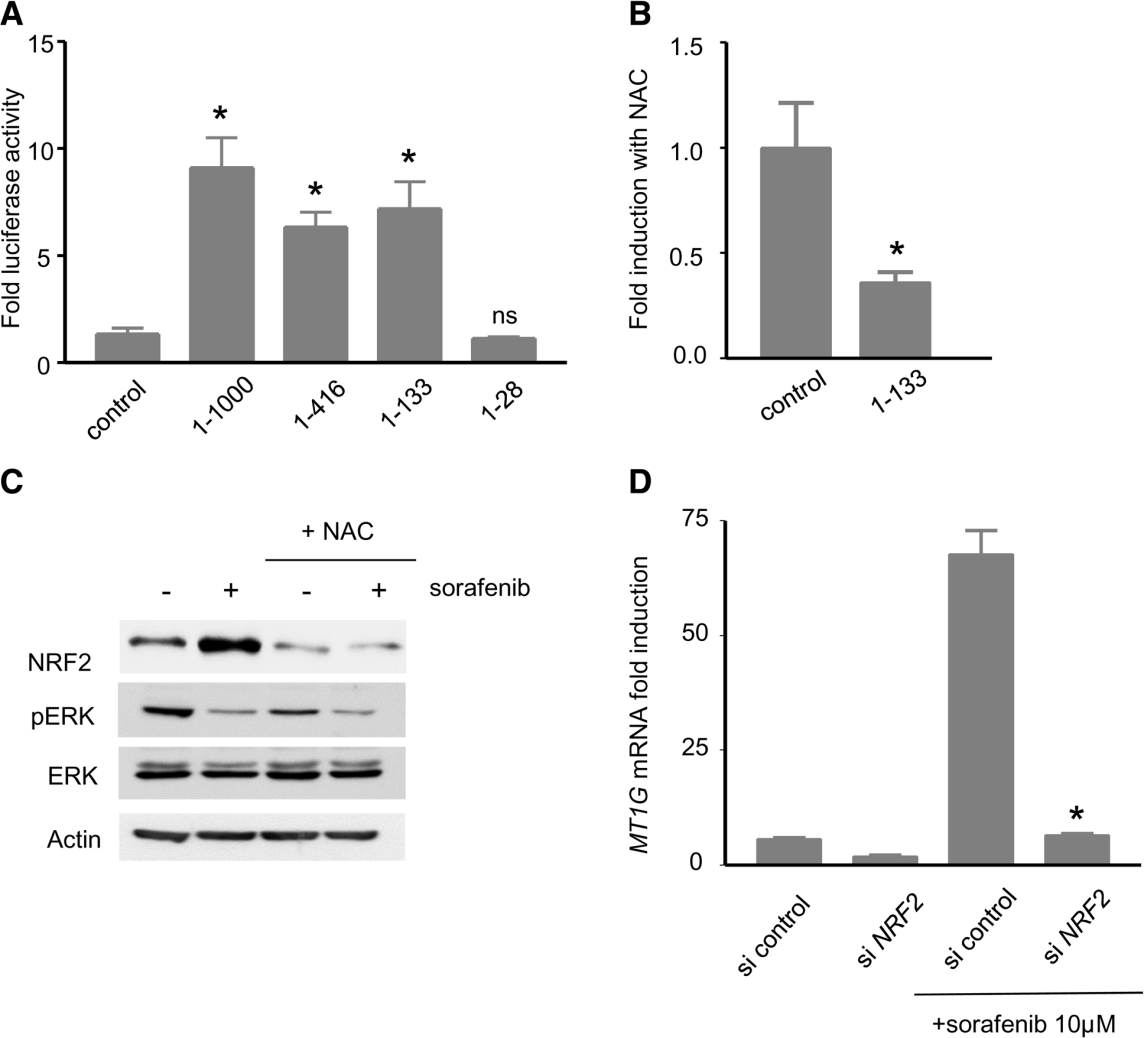
*Analysis of the promoter of MT1G and its induction by sorafenib*. **a** Luciferase activity from different constructions derived from the promoter of MT1G. The constructions -1000, -416, -133, -28 nt and control (empty vector) were transfected into Huh7 cells, exposed to sorafenib 10 μM for 18 h. *: *p* < 0.05 compared to control. n.s.: non-significant compared to control. **b** analysis of the effect of NAC on the transcriptional activation of the 1-133 region of *MT1G* promoter by sorafenib. Luciferase activity was evaluated from Huh7 cells transfected with the 1-133 construction and the empty vector. Cells were preincubated with 10 mM NAC shortly before transfection and luciferase activity was measured after 18 h. The values of luciferase activity are normalized to the conditions without NAC, and expressed as a fold-induction with NAC. *: *p* < 0.05 compared to control. **c** Western blot with NRF2, ERK, pERK and Actin antibodies on Huh7 cells treated for 18 h with sorafenib 10 μM and/or NAC 10 mM (preincubated 1 h). **d** *MT1G* mRNA levels were analysed in Huh7 cells transfected with control siRNA *vs* siRNA directed against NRF2, and exposed to sorafenib 10 μM for 18 h as indicated. *: *p* < 0.05 compared to control siRNA treated with sorafenib

In order to identify the minimal region of *MT1G* promoter able to confer sorafenib inducibility, four constructs encompassing various portions of the *MT1G* promoter were introduced upstream of the gene encoding the enzyme luciferase. Each reporter construct was transfected into Huh7 cells, and cells were subsequently exposed to sorafenib (Fig. 3a). All constructions encompassing 133 nucleotides upstream of the transcription starting site conferred a strong, close to 10-fold inducibility by sorafenib (Fig. 3a). Interestingly, this region of the promoter contains an Antioxidant Response Element (ARE) [21]. We verified that this sequence confers redox control over the transcription of the *MT1G* gene by testing the effect of NAC on luciferase activity measured with the 1-133 construct (Fig. 3b). Addition of NAC abolished the increase in Luciferase activity observed with sorafenib on the 1-133 construct, showing a redox-dependent regulation of this region of the *MT1G* promoter.

Antioxidant Response Elements (ARE) are cis-acting elements that are recognized by the transcription factor NRF2, reported to play an essential role in the coordination of the transcriptional response of eukaryotic cells exposed to conditions of oxidative stress [22]. Interestingly, sorafenib greatly increased the expression levels of NRF2 in Huh7 cells, and NAC abolished this increase (Fig. 3c). In order to definitely establish the role of NRF2 in the regulation of the *MT1G* gene in cells exposed to sorafenib, we used RNA interference against NRF2. Small interfering RNA (siRNA) directed against *NF2E2* was applied to Huh7 cells and was found to reduce the levels of NRF2 to 50 % of its control values (data not shown). Compared to control conditions, it prevented the induction of *MT1G* observed upon exposure of Huh7 cells to sorafenib (10 μM, 18 h) (Fig. 3d). The findings demonstrated a NRF2-dependent regulation of the transcription of the *MT1G* gene in cancer cells exposed to sorafenib.

### Role of MT1G in the response of HCC cells to sorafenib

**Fig. 4:**
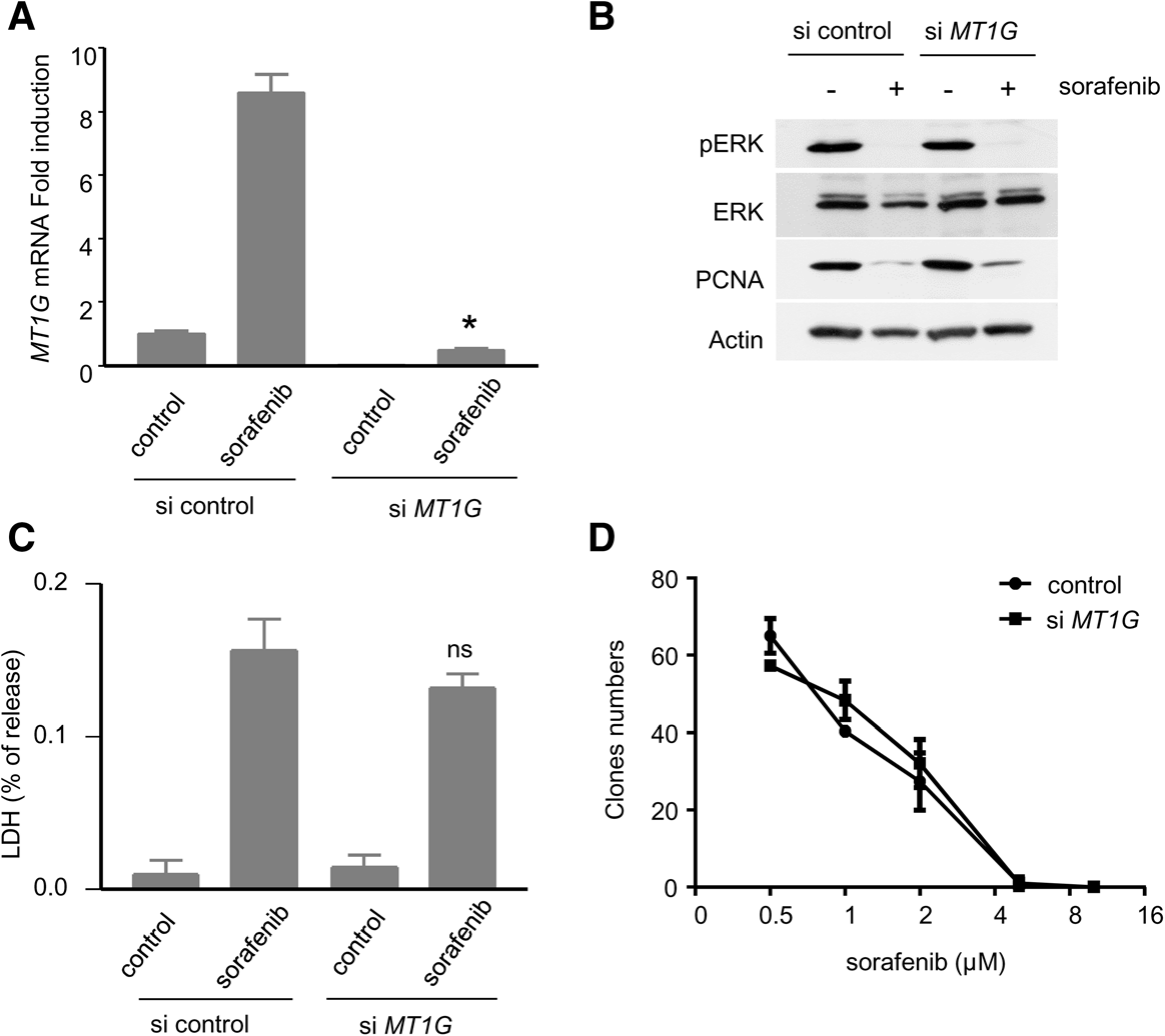
*Role of MT1G in Huh7 cells*. **a** Induction of *MT1G* mRNA in Huh7 cells transfected with MT1G siRNA and control siRNA, exposed to sorafenib (10 μM, 18 h). *: *p* < 0.05 compared to control siRNA treated with sorafenib. **b** Analysis of the effects of sorafenib (10 μM, 18 h) on its main target kinases (as reflected by the activation levels of the RAF-MEK-ERK cascade) in Huh7 cells transfected with control siRNA or siRNA directed against *MT1G*. **c**: Ferroptosis induction by sorafenib. A value of % released LDH was calculated for each condition in Huh7 transfected with control siRNA *vs MT1G* siRNA, and cells were treated with sorafenib (10 μM, 18 h) as indicated. ns: non-significant compared to control siRNA exposed to sorafenib. **d** Clonogenic growth of Huh7 cells exposed to sorafenib applied at different concentrations (0.5, 1, 2, 5 and 10 μm) after transfection with the indicated siRNA

In order to explore the significance of MT1 induction in cancer cells exposed to sorafenib, we knocked-down the expression of *MT1G* in Huh7 cells (Fig. 4a). A siRNA that targets *MT1G* was found to abolish the induction of the corresponding mRNA in Huh7 cells exposed to sorafenib (Fig. 4a). In the corresponding conditions, we did not detect any effect of *MT1G* on the expression levels of phosphorylated ERK1/2, reflecting the impact of sorafenib on the RAF kinases (Fig. 4b). We also did not find any difference in the expression levels of the protein PCNA (Proliferating cell nuclear antigen) or caspases, suggesting that neither cell proliferation nor apoptosis levels were significantly impacted by *MT1G* (Fig. 4b, data not shown). A direct measurement of the levels of ferroptosis induced by sorafenib, performed by comparing the % LDH released in control- or si*MT1G*-treated Huh7 cells also failed to reveal any significant difference (Fig. 4c). Cells with low levels of *MT1G* also displayed similar properties in clonogenic growth upon exposure to increasing concentrations of sorafenib (Fig. 4d), further suggesting the lack of an important modulatory effect of *MT1G* on the response of these cells to sorafenib.

### MT1 as a clinically-applicable biomarker

**Fig. 5:**
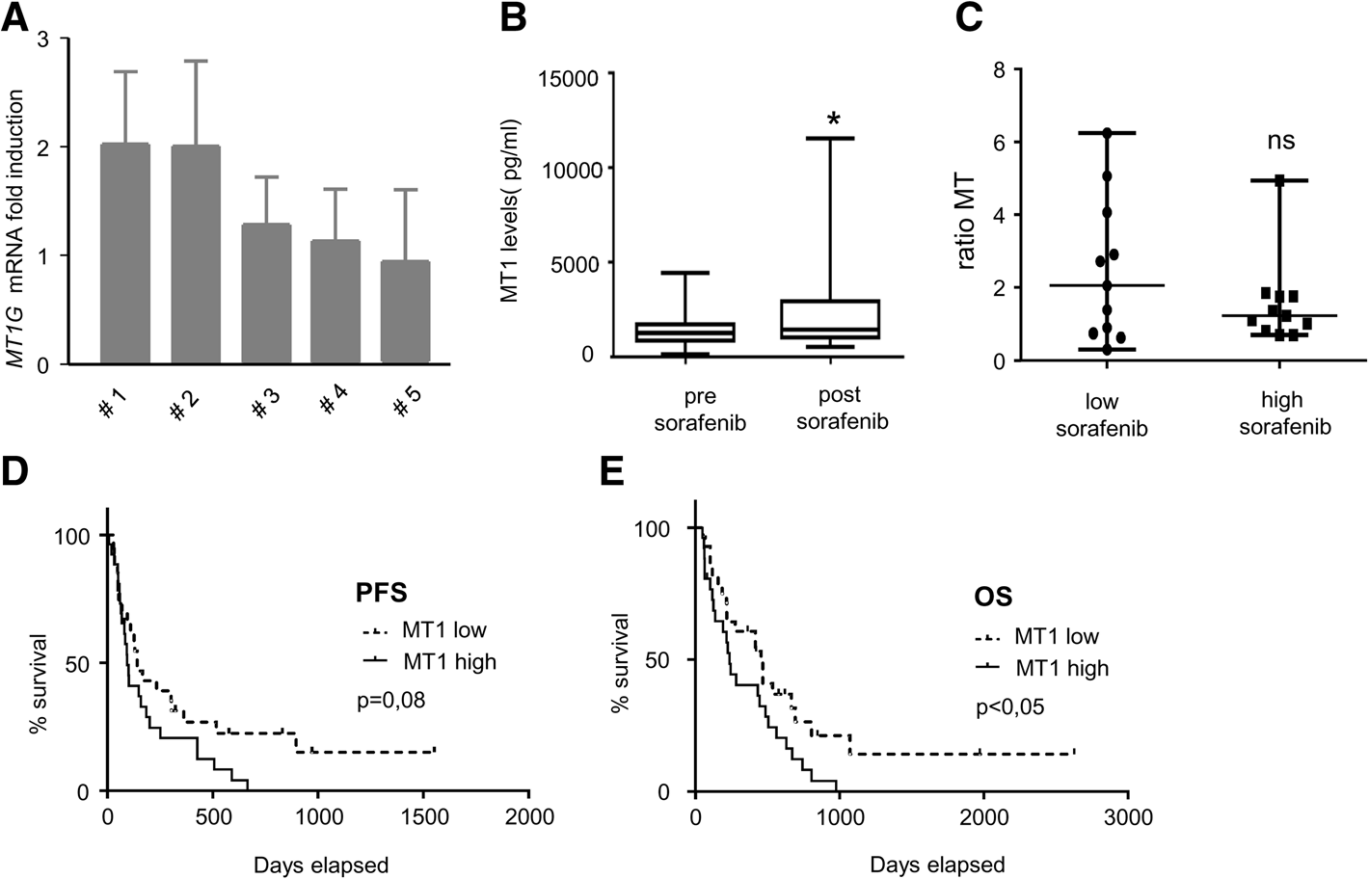
*MT1 as a clinically-applicable biomarker*. **a**: Evaluation of *MT1G* induction in five HCC tumour explants. Tumour slices were prepared from five surgically-resected HCC and maintained in culture for 18 h in control conditions or in the presence of sorafenib (10 μM). The levels of *MT1G* mRNA were evaluated by QPCR, and the values were used to calculate a ratio. **b** Levels of MT1 were measured in the sera of 20 patients with advanced HCC and treated with sorafenib. Serum MT1 values are presented before and after sorafenib treatment. * indicates *p* < 0.05 between the two series using paired Wilcoxon test. **c** MT1 concentration in the patient population with low serum concentrations of sorafenib (*i.e.* below the median) and the patient population with high serum concentrations of sorafenib (*i.e.* above the median). The graph is based on the calculated ratio of the serum values of MT1 measured after sorafenib treatment and MT1 before sorafenib treatment. n.s.: indicates a lack of significant difference between the two groups. **d** Kaplan-Meier analysis of the progression-free survival (PFS) of HCC patients receiving sorafenib. Patients were divided into two groups: one with the lowest and one with the highest induction of MT1 upon sorafenib treatment (based on the calculation of the ratio of MT1 before/MT1 after sorafenib treatment, and using the median value as cut-off). **e** Kaplan-Meier analysis of the overall survival (OS) of HCC patients receiving sorafenib

In order to explore the clinical relevance of our findings, we carried out short-term culture of tumour explants prepared from five HCC tumours (Fig. 5a) [23]. Tumour samples were obtained from five patients undergoing surgical resection with curative intent (see Additional file 1: Table S2). The tumours were prepared as thin slices and maintained for 18 h in the presence of sorafenib in order to examine its effect on *MT1G* mRNA expression levels. A more than two-fold increase in *MT1G* mRNA upon exposure to sorafenib (10 μM, 18 h) was seen in two out of the five HCC tumours (Fig. 5a). Next, we measured the protein levels of MT1 in the serum of two cohorts of patients with HCC. The clinical characteristics of the patients are presented in the Additional file 1: Table S3. The first cohort consisted of 20 patients with HCC treated with sorafenib (Fig. 5b) [24]. For each patient, serum samples were obtained before and 4 to 24 weeks after the onset of treatment with sorafenib. Using an ELISA kit that measures the presence of all isoforms of MT1, we found that serum levels of MT1 significantly increased in the serum of patients with HCC treated with sorafenib (median value before sorafenib: 1225 pg/mL; median value after sorafenib: 1353 pg/mL, *p* < 0.05 using paired Wilcoxon test). At the individual level, 7 out of the 20 patients in this exploratory cohort presented a more than three-fold increase in MT1 upon sorafenib treatment. In order to examine the possibility that the induction of MT1 might partially reflect the exposure of tumour cells to sorafenib, we examined the serum trough concentration of sorafenib measured in individual patients (Fig. 5c). Serum concentrations of sorafenib were highly heterogeneous among the patients, with a more than 80-fold difference between the minimum and the maximum concentrations (min. concentration: 0.5 μM; max.: 41.3 μM; median: 7.1 μM). However, we found no significant difference in terms of induction of MT1 in the population with low concentrations of sorafenib (*i.e.* the patients with serum concentrations below the median) and the population with high concentrations of sorafenib (*i.e.* above the median): in these two groups, the median values calculated for the ratio MT1 after/before sorafenib were 2.05 and 1.22, respectively (*p* = 0.40 using Mann-Whitney).

To examine the clinical impact of MT1, serum levels of MT1 and overall survival (OS) and progression-free survival (PFS) were analysed in a second cohort of 55 patients with HCC treated with sorafenib. We performed a Kaplan-Meier analysis comparing PFS and OS in the groups with the lowest and highest induction of MT1 upon sorafenib treatment (based on the calculation of the ratio of MT1 before/MT1 after sorafenib treatment, and using the median value as cut-off) (Fig. 5d, e). This analysis revealed that the group of patients presenting with the highest induction of MT1 had a tendency toward a reduced PFS compared to the group with the lowest induction (Median PFS: 93 days *vs* 136 days, *p* = 0.08 using Log-rank test, Fig. 5d). Strikingly, the group of patients presenting with the highest induction of MT1 had a significantly reduced OS compared to the group with the lowest induction (Median overall survival: 237 days *vs* 456 days, respectively; *p* < 0.05 using Log-rank test) (Fig. 5e). No statistically-significant difference were found between the two groups in terms of clinical characteristics (Age, Sex, Child Pugh score, etiology of cirrhosis, or presence of metastasis) (data not shown). We concluded that high induction of MT1 in the serum was indicative of poor prognosis in HCC patients treated with sorafenib.

## Discussion

In the present study, we identify MT1 as a potential new biomarker reflecting the impact of sorafenib on the redox metabolism of cancer cells. We confirm and extend the conclusions of the previous studies that revealed that sorafenib is able to induce oxidative stress in various cancer cells, in vitro and in animal models [4–9]. Using a combination of pharmacological and molecular approaches we showed that the transcription factor NRF2, an essential regulator of redox metabolism in eukaryotic cells [22], is activated upon exposure of cancer cells to sorafenib. We identified a region of 133 bp, located immediately upstream of the transcription start site in the promoter of *MT1G*, that constitutes the minimal region able to confer transcriptional inducibility by sorafenib. This region of the promoter contains an ARE, known to constitute a recognition site for NRF2 [21, 22]. In full agreement with these observations, we showed that pharmacological anti-oxidants, such as NAC, or the knock-down of NRF2 prevented the induction of *MT1G* induced by sorafenib. Interestingly, we observed no induction of *MT1G* in primary human hepatocytes, and a striking specificity was also observed when sorafenib was compared with a panel of other clinically-approved kinase inhibitors in terms of ability to induce *MT1G*. Our study therefore identifies *MT1G* as a gene whose expression levels reflect the specific effect of sorafenib on the redox metabolism of cancer cells.

Redox metabolism of cancer cells is an essential facet of tumour physiology and a major determinant of cancer cell fate during tumour development and in the therapeutic context [24–26]. The NRF2 transcription factor is an essential coordinator for the regulation of the expression of the main genes and molecules with antioxidant function in eukaryotic cells [22]. Exome sequencing of human HCC tumours revealed the presence of somatic mutations that activate NRF2 in a substantial fraction of HCC [27–29]. Recently, the NRF2 status was directly reported to modulate the response of HCC cells to sorafenib [7]. In HCC cells with low levels of NRF2 achieved through RNA interference, sorafenib was found to induce ferroptosis with a higher efficacy [7]. These findings suggest that the NRF2 status could be an important determinant of the response of sorafenib, though it is presently partially unclear which target genes could mediate this effect of NRF2. In the present study, we did not observe a modulatory role of *MT1G* on the clonogenic growth of HCC cells or their susceptibility to ferroptosis, a new form or regulated oxidative necrosis. At this stage, it must however be pointed out that functional redundancy between the isoforms of MT1, and the choice of the genetic background used to explore the role of MT1G may explain the lack of a phenotype. More studies are required to address the role of MT1 in solid tumours, especially in the context of therapeutic targeting.

Our analyses performed using tumour explants and serum samples obtained from cancer patients treated with sorafenib demonstrate the clinical applicability of MT1 as a biomarker. Compared to the previous report by Coriat et al. presenting a first clinical analysis centred on the measurement of serum levels of AOPP [4], our study brings three important additional pieces of information: i) Firstly, the measurement of MT1 provides a more specific and standardizable analysis for the study of the effects of sorafenib on the redox metabolism of cancer cells than AOPP; ii) Secondly, our study highlights the existence of important individual heterogeneity in terms of the ability of sorafenib to activate the transcription of the MT1 genes. Tumour-intrinsic determinants at least partially account for this heterogeneity, since we observed that human cancer cell lines and individual tumour explants present great differences in their induction of *MT1G* mRNA upon exposure to sorafenib. Based on our results demonstrating the role of oxidative metabolism in the regulation of *MT1G*, it is tempting to speculate that this patient heterogeneity might at least partially reflect individual differences in the effects of sorafenib on the oxidative metabolism of cancer cells in different tumours. Whether these differences are related to tumour genotype and the presence of somatic mutations of NRF2 [27–29] is an interesting possibility, but further studies are required to address these possibilities; iii) thirdly, HCC patients with the highest induction of MT1 have reduced overall survival under sorafenib treatment, suggesting the possibility that the alteration of the redox metabolism of cancer cells might be detrimental to HCC patients receiving sorafenib. These conclusions might at first-look appear to be in contrast with the work by Coriat et al. [4] and the in vitro studies that recently highlighted the remarkable ability of sorafenib to induce ferroptosis [5–7]. A possible explanation might be that the serum levels of AOPP and the markers of ferroptosis reflect intense oxidative damage induced by sorafenib, while the present study explored the transcriptional regulation of *MT1* in cancer cells, and therefore the impact of sorafenib at lower, sub-cytotoxic concentrations. Whether such an effect of sorafenib could limit its anti-oncogenic activity, perhaps by activating specific oncogenic signal transduction cascades in tumour cells [30, 31], is an interesting possibility that deserves further study.

## Conclusions

Our findings further establish the remarkable ability of sorafenib to alter the redox metabolism of some, but not all cancer cells. We show that MT1 constitute a biomarker adapted for exploring the impact of sorafenib on the redox metabolism of cancer cells.

## Methods

### Cell culture and reagents

A list of all cancer cell lines and primary cells used in this manuscript is provided in a Supplementary Materials and methods section [see Additional file 2]. Sorafenib and all kinase inhibitors were purchased from Selleck chemicals.

### Microarray experiments

Samples were prepared from 500 ng total RNA using the GeneChip WTPLUS Reagent kit protocol and hybridized on Affymetrix four-array strips (HuGene 2.1 ST array strips). After hybridization (19 h at 48 °C) on the Affymetrix GeneAtlas hybridization station, strips were washed and imaged on the GeneAtlas imaging station. Data were normalized using the expression Console software using default RMA-sketch normalization. Normalized files were analyzed using TAC software (Affymetrix) with default settings. A summary of the results is presented in Additional file 1: Table S1.

### RNA interference

Small interfering RNA directed against *MT1G*, *NFE2L2* and silencer negative control were purchased from Life technologies and were transfected using the siPORT-neoFX reagent (Life Technologies), according to the manufacturer’s instructions. The sequence of the siRNA targeting *MT1G* was 5′-GCUCCCAAGUACAAAUAGAtt-3′ and was specific to this isoform.

### Analysis of the activity of the MT1G promoter by luminescence

DNA constructs encompassing various portions of the human *MT1G* promoter (position -1000 to -28 from the transcription starting site) were inserted in the pGL3-Basic vector, encoding firefly (Photinus pyralis) luciferase (Promega). Transfection was performed using the Genjet reagent, as indicated by the provider (Tebu-Bio). Luciferase activity was measured on a CentroLB 960 Microplate Luminometer (Berthold). All values were normalized according to the protein concentration of the extracts, and a fold induction was calculated by comparing control vs sorafenib conditions.

### Short-term culture of HCC explants

We have previously reported the use of short-term culture of tumour explants for the exploration of the individual sensitivity of HCC tumours to sorafenib [23]. Tumour samples were obtained from five patients undergoing surgical resection with curative intent performed between January 2015 and July 2015 in Amiens University Hospital (France). The corresponding protocol was approved by the Comité de Protection des Personnes Nord-Ouest (CPP NO 2009/14). Patient consent was obtained for the use of resected tumours. The summary of the tumour characteristics can be found in the Additional file 1: Table S2.

### Determination of serum MT1 levels

Frozen serum samples from two cohorts of patients with HCC receiving sorafenib were used in this study. Details regarding the patients recruited can be found in a previous study [24] and in the Additional file 2 and Additional file 1: Table S3. Serum levels of MT1 were determined using a sandwich-based ELISA kit (Cusabio, CSB-E09060h).

### Statistical analyses

Student’s *t*-test was used for the interpretation of the experiments performed on cells. Paired Wilcoxon test, Mann-Whitney test, and Kaplan Meier were used as indicated for analyses performed on the patient cohorts. In each case, a value of *p* < 0.05 was considered as threshold for significance.

## Ethics approval and consent to participate

This study was conducted in compliance with the French legislation and the declaration of Helsinki regarding ethical principles for medical research involving human subjects. The use of surgically-resected tumours for research purposes in the laboratory of Biochemistry of the University Hospital of Amiens was approved by the Comité de Protection des Personnes Nord-Ouest (CPP NO ref. 2009/14). No samples were obtained from any patients that were minor or physically or mentally unable to understand and give their consent to the use of surgical samples.

## Availability of data and materials

The dataset supporting the conclusion of this article are available in the Gene Expression Omnibus (GEO NCBI) public database under accession number GSE75620 [http://www.ncbi.nlm.nih.gov/geo/query/acc.cgi?acc=GSE75620].

## Additional files

Additional file 1: Figure S1. Sorafenib increases the expression levels of MT1B in Huh7 cells. **Figure S2**: Pharmacological antioxidants prevent the induction of MT1B induced by sorafenib. **Table S1**: List of genes upregulated in Huh7 cells exposed to sorafenib (10 μM) for 9 h. **Table S2**: Summary of the characteristics of the hepatocellular carcinoma tumours used for short-term culture of tumour explants. Table S3: Summary of the clinical characteristics of HCC patients in the two cohorts. (DOC 185 kb)

Additional file 2: Supplementary materials and methods. (DOC 44 kb)

## Abbreviations

AOPP: advanced oxidation products of proteins
ARE: Antioxidant Response Element
GSH: Glutathione
HCC: hepatocellular carcinoma
MT1: Metallothionein-1
NAC: N-Acetyl-Cysteine
NRF2: Nuclear factor erythroid 2 [NFE2]-related factor 2
PFS: progression-free survival
PHH: primary human hepatocytes
OS: overall survival
ROS: reactive oxygen species
siRNA: small interfering RNA
