# Supplementary material for: Metallothionein-1 as a biomarker of altered redox metabolism in hepatocellular carcinoma cells exposed to sorafenib
Source: Mol Cancer. 2016 May 16;15:38. doi: 10.1186/s12943-016-0526-2 (PMC4894370; doi:10.1186/s12943-016-0526-2)
Supplement: Supplementary file 1 — Sorafenib increases the expression levels of MT1B in Huh7 cells. Figure S2: Pharmacological antioxidants prevent the induction of MT1B induced by sorafenib. Table S1: List of genes upregulated in Huh7 cells exposed to sorafenib (10 μM) for 9 h. Table S2: Summary of the characteristics of the hepatocellular carcinoma tumours used for short-term culture of tumour explants. Table S3: Summary of the clinical characteristics of HCC patients in the two cohorts. (DOC 185 kb) [file 12943_2016_526_MOESM1_ESM.doc]

*Supplementary Figures & Tables*

**Metallothionein-1 as a biomarker of altered redox metabolism in hepatocellular carcinoma cells exposed to sorafenib**

*Houessinon et al.*

***Suppl. Fig.*** ***1****: Sorafenib increases the expression levels of MT1B in Huh7 cells*

***Suppl. Fig. 2****:* *Pharmacological antioxidants prevent the induction of MT1B induced by sorafenib.*

***Suppl. Table 1****: List of genes upregulated in Huh7 cells exposed to sorafenib (10 µM) for 9h*

***Suppl. Table 2****: Summary of the characteristics of the hepatocellular carcinoma tumours used for short-term culture of tumour explants*

***Suppl. Table 3****: Summary of the clinical characteristics of HCC patients in the two cohorts*

**
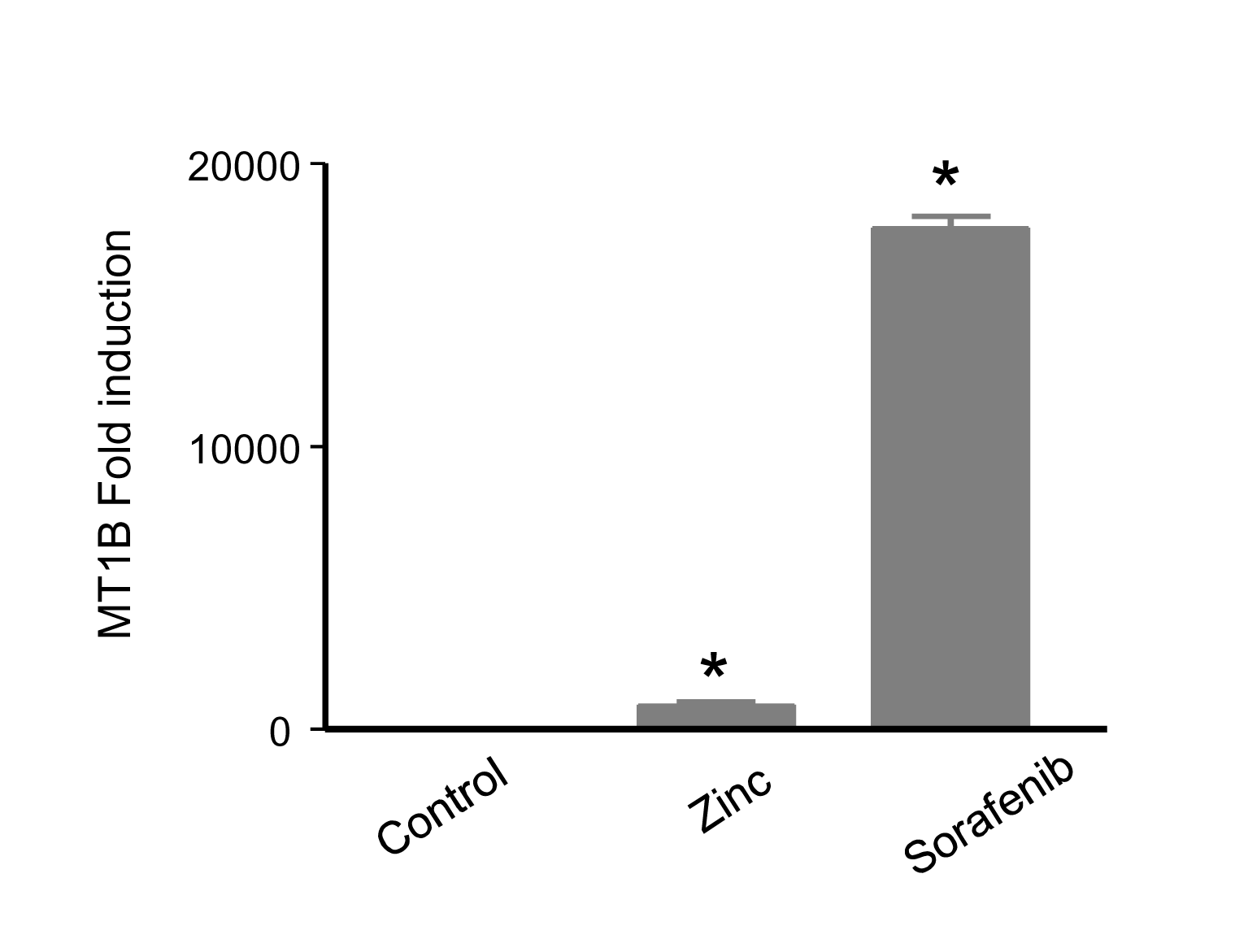
**

**Suppl. Fig. 1: Sorafenib increases the expression levels of MT1B in Huh7 cells.** Huh7 cells were exposed to sorafenib (10 µM) or ZnCl_2_ (100 µM) for 18h. mRNA levels of MT1B were evaluated by QPCR. The results are presented as fold-increase over control conditions, after normalization to GAPDH mRNA levels, and are based on three experiments. *: p<0.05 compared to control.

*
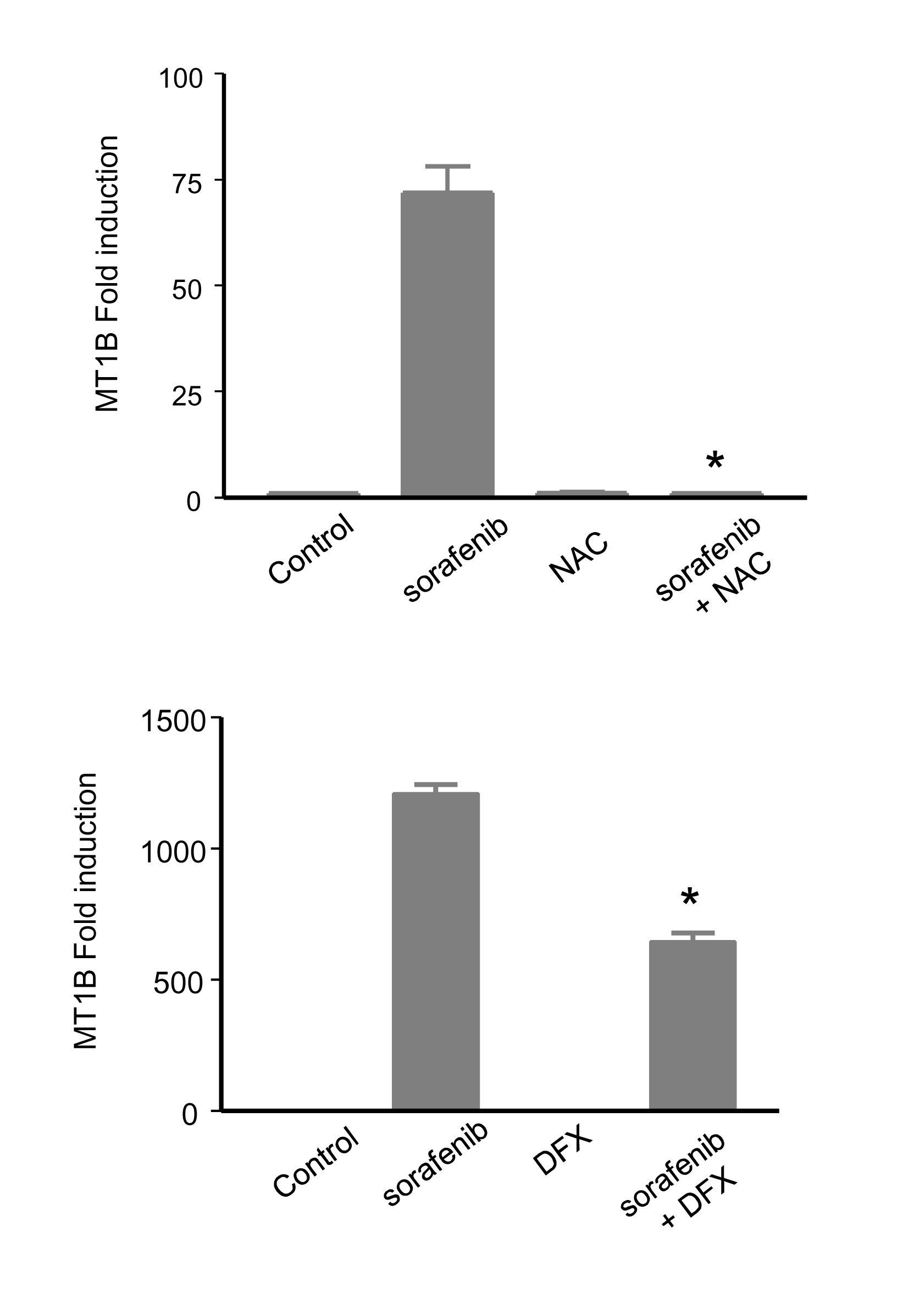
*

**Suppl. Fig. 2. Pharmacological antioxidants prevent the induction of MT1B induced by sorafenib.** MT1B mRNA induction in Huh7 cells treated with sorafenib 10µM and DFX 100 µM (preincubated 1 hour) or NAC 10 mM (preincubated 1 hour), and maintained for 18 hours. *: p<0.05 compared to sorafenib alone.

**Suppl. Table 1 : Summary of the genes upregulated in Huh7 cells upon sorafenib treatment (10 µM for 9h) in the microarray assay.**

|  | **Transcript Cluster ID** | | **Fold Change** | | **ANOVA p-value** | | **FDR p-value** | | **Gene Symbol** | | **Description** | |
| --- | --- | --- | --- | --- | --- | --- | --- | --- | --- | --- | --- | --- |
| 16692636 | | -3.00 | | 0.028615 | | 0.473556 | | HIST2H2AB | | histone cluster 2, H2ab | |  |
| 16819257 | | -3.08 | | 0.011922 | | 0.423393 | | MT1H | | metallothionein 1H | |  |
| 16855127 | | -3.13 | | 0.014584 | | 0.436252 | | SMAD7 | | SMAD family member 7; NULL | |  |
| 16819224 | | -3.13 | | 0.009018 | | 0.412728 | | MT1M | | metallothionein 1M | |  |
| 16694359 | | -3.14 | | 0.008425 | | 0.412728 | | SCARNA4 | | small Cajal body-specific RNA 4 | |  |
| 16858710 | | -3.14 | | 0.003674 | | 0.406778 | | JUNB | | jun B proto-oncogene | |  |
| 16786650 | | -3.16 | | 0.011000 | | 0.420556 | | RNA5SP387 | | RNA, 5S ribosomal pseudogene 387 | |  |
| 16670918 | | -3.17 | | 0.003143 | | 0.406778 | | MIR554 | | microRNA 554 | |  |
| 17016486 | | -3.17 | | 0.026040 | | 0.472893 | | HIST1H2BL | | histone cluster 1, H2bl | |  |
| 16968077 | | -3.24 | | 0.046930 | | 0.506861 | | CCNG2 | | cyclin G2 | |  |
| 16863877 | | -3.24 | | 0.006025 | | 0.406778 | | PPP1R15A | | protein phosphatase 1, regulatory subunit 15A | |  |
| 17078452 | | -3.25 | | 0.006827 | | 0.406778 | | HEY1 | | hairy/enhancer-of-split related with YRPW motif 1; NULL | |  |
| 16836528 | | -3.27 | | 0.022576 | | 0.468840 | | YPEL2 | | yippee-like 2 (Drosophila); NULL | |  |
| 16678496 | | -3.28 | | 0.021975 | | 0.468840 | | HIST3H2BB | | histone cluster 3, H2bb | |  |
| 16819217 | | -3.41 | | 0.000409 | | 0.372493 | | MT1E | | metallothionein 1E | |  |
| 17002052 | | -3.65 | | 0.013580 | | 0.428187 | | FAXDC2; C5orf4 | | fatty acid hydroxylase domain containing 2; NULL | |  |
| 16826738 | | -3.69 | | 0.014956 | | 0.436403 | | MT1G | | metallothionein 1G | |  |
| 16698466 | | -3.84 | | 0.001987 | | 0.406778 | | NUAK2 | | NUAK family, SNF1-like kinase, 2 | |  |
| 16937024 | | -4.08 | | 0.002478 | | 0.406778 | | BHLHE40 | | basic helix-loop-helix family, member e40 | |  |
| 16926200 | | -4.08 | | 0.004172 | | 0.406778 | | SIK1 | | salt-inducible kinase 1 | |  |
| 16677278 | | -4.20 | | 0.005408 | | 0.406778 | | ATF3 | | activating transcription factor 3; NULL | |  |
| 16974315 | | -4.44 | | 0.005256 | | 0.406778 | | RNA5SP155 | | RNA, 5S ribosomal pseudogene 155 | |  |
| 16825371 | | -4.46 | | 0.000664 | | 0.372493 | | NUPR1 | | nuclear protein, transcriptional regulator, 1 | |  |
| 16671642 | | -4.48 | | 0.015127 | | 0.437456 | | EFNA1 | | ephrin-A1; NULL | |  |
| 16849635 | | -4.55 | | 0.004455 | | 0.406778 | | CBX4 | | chromobox homolog 4 | |  |
| 16819247 | | -4.59 | | 0.005771 | | 0.406778 | | MT1B | | metallothionein 1B | |  |
| 16819213 | | -4.77 | | 0.003086 | | 0.406778 | | MT1L; NUTF2 | | metallothionein 1L (gene/pseudogene); nuclear transport factor 2 | |  |
| 16974830 | | -4.87 | | 0.000972 | | 0.379904 | | PPARGC1A | | peroxisome proliferator-activated receptor gamma, coactivator 1 alpha; NULL | |  |
| 17012946 | | -5.42 | | 0.000066 | | 0.372493 | | TNFAIP3 | | tumor necrosis factor, alpha-induced protein 3 | |  |
| 16858210 | | -5.44 | | 0.004368 | | 0.406778 | | SLC44A2 | | solute carrier family 44, member 2; NULL | |  |
| 16764053 | | -5.55 | | 0.003253 | | 0.406778 | | RND1 | | Rho family GTPase 1; NULL | |  |
| 16705961 | | -5.65 | | 0.019924 | | 0.460900 | | DDIT4 | | DNA-damage-inducible transcript 4 | |  |
| 16766578 | | -5.69 | | 0.006742 | | 0.406778 | | DDIT3 | | DNA-damage-inducible transcript 3 | |  |
| 17104049 | | -5.87 | | 0.019770 | | 0.460870 | | SNORA11; MAGED2 | | small nucleolar RNA, H/ACA box 11; melanoma antigen family D, 2 | |  |
| 16671632 | | -6.33 | | 0.015089 | | 0.437456 | | EFNA3; OTTHUMG00000161134; RP11-540D14.8 | | ephrin-A3; NULL | |  |
| 16859800 | | -9.86 | | 0.007518 | | 0.411527 | | MIR3189 | | microRNA 3189 | |  |
| 16856803 | | -12.43 | | 0.000746 | | 0.372749 | | GADD45B | | growth arrest and DNA-damage-inducible, beta | |  |

Genes are ranked according to the fold-induction upon exposure to sorafenib (10 µM, 9h).

The genes of the metallothionein-1 family are shown in red.

**Suppl. Table 2: Clinical characteristics of the HCC tumours used for short-term culture of tumour explants**

**Tumour id. #1 #2 #3 #4 #5**

Gender (M / F) M M M M M

Age (Years) 71 66 72 59 59

Tumour size (cm) 4 3 4 9 5

Cirrhosis no Child A Child A no Child A

AFP (ng/mL) 2.3 11.6 - 3200 21.7

**Suppl. Table 3: Summary of the clinical characteristics of HCC patients in the two cohorts**

**Cohort #1 Cohort #2**

(n=20) (n=55)

Gender (M / F) 17 / 3 40 / 15

Age (Years, median) 68 61

Child-Pugh

0 2 12

A 14 41

B 6 2

Main etiology of cirrhosis

Alcohol 8 4

NASH 0 11

Virus 6 21

Mixed 5 4

Unknown 1 10
