# Supplementary material for: Metallothionein-1 as a biomarker of altered redox metabolism in hepatocellular carcinoma cells exposed to sorafenib
Source: Mol Cancer. 2016 May 16;15:38. doi: 10.1186/s12943-016-0526-2 (PMC4894370; doi:10.1186/s12943-016-0526-2)
Supplement: Supplementary file 2 — Supplementary materials and methods. (DOC 44 kb) [file 12943_2016_526_MOESM2_ESM.doc]

*Supplementary Materials and Methods*

**Metallothionein-1 as a biomarker of altered redox metabolism in hepatocellular carcinoma cells exposed to sorafenib**

*Houessinon et al.*

*Cell culture.* Huh7 cells were a kind gift from Dr. Wychowski (Institut de Biologie de Lille, France). The cell lines Hep3B, BxPC3, ACHN, NCIH, HCT116 and PANC1 were purchased from ATCC. Primary Human Hepatocytes (PHH) were prepared using samples from partial hepatectomy performed in Amiens University hospital, as described in Helle *et al.* (2013). Cells were cultured in Dulbecco’s Modified Eagle’s Medium (Sigma, Saint Quentin Fallavier, France) supplemented with 10% fetal calf serum (Jacques Boy, Reims, France), 2 mM glutamine, and penicillin / Streptomycin.

*Quantitative PCR*. Total RNA was extracted using RNA easy Mini Kit (Qiagen) and reverse-transcribed using High Capacity cDNA Reverse Transcription kit and random hexamer (Applied). Amplification was performed with the TaqMan Universal PCR master Mix on an ABI 7900HT Sequence Detection System (Applied) using primers and probe sets for all isoforms of human MT1 and GAPDH (TaqMan Gene Expression Assay, Applied).

*Western blots.* Cell extracts were prepared in RIPA buffer, and loaded on SDS-PAGE, transferred to nitrocellulose membranes and immunoblotted using standard procedures. The ECL reaction was used for revelation (Galmiche et al., 2008). Antibodies raised against NRF2, the Proliferating Cell-Nuclear Antigen (PCNA) and MT1 were from Abcam. Rabbit antibodies directed against Extracellular Regulated Kinase 1/2 (ERK1/2) and ERK1/2 phosphorylated on Thr202/Tyr204 (p-ERK) were from Cell Signaling. Mouse anti-β actin was from Sigma.

*Clonogenicity and Cell viability assay.* Clonogenic growth was measured as previously described (Galmiche et al., 2010). Briefly, 200 viable Huh7 cells were seeded and exposed to the indicated conditions. After 15 days of culture, the clones were stained with Giemsa and a blind count was performed. Lactate Dehydrogenase (LDH) released in the culture medium was measured with the CytoTox 96 Non-Radioactive Cytotoxicity Assay kit (Promega, Charbonniere, France). The results are expressed as % of released LDH, taking complete cell extract as 100%.

*Sorafenib quantification assay*. Sorafenib trough concentrations were assayed by liquid chromatography-tandem mass spectrometry. Chromatographic separation was performed with a kinetex C18 column (100x4.6 mm; 2.6 μm particle size; Prominence; Shimadzu, Marne La Vallée, France). Detection was performed with a triple quadrupole mass spectrometer (3200 QTRAP; ABsciex, Les Ulis, France).

Patient sera. Frozen serum samples from a first cohort of 20 patients with advanced HCC were provided by the Paris-Seine-Saint-Denis University Hospital liver Biobank. The patients were recruited in this institution between October 2010 and May 2013. Basal serum samples were collected from these patients before the treatment, and a second serum sample was collected 4 to 24 weeks after the onset of treatment with sorafenib. A second cohort consisting of 55 patients with advanced HCC was obtained from Cochin Hospital (Paris, France). Patients were enrolled in the study and treated with sorafenib using the validated dose of 400 mg twice daily until there was evidence of disease progression. Patients were classified as having advanced disease if they were not eligible for surgery or had disease progression after surgical or locoregional therapy. Sorafenib was administered at 50% of the planned dose if any severe adverse event related to the drug occurred, and in frail patients 1. Treatment interruptions and up to two dose reductions (first to 400 mg once daily, and then to 400 mg every 2 days) were applied in case of drug-related adverse effects. A retrospective review of electronic medical records of patients with advanced HCC treated with sorafenib was carried out. Tumour evaluation was performed every three months during treatment, according to the RECIST criteria. Progression-free survival (PFS) was measured from the date of first treatment administration to the date of evidence of progression or death. Overall survival (OS) was measured from the date of first treatment administration to the date of death or last follow-up. Sera were collected from each patient 7 days before and 14 days after sorafenib start. The clinical and biological characteristics of the patients from both cohorts are summarized in Supplementary Table 3.

**References**

Galmiche A, et al. Isoform-specific interaction of C-RAF with mitochondria. J Biol Chem. 2008; 283: 14857-66.

Helle F, et al. Permissivity of primary human hepatocytes and different hepatoma cell lines to cell culture adapted hepatitis C virus. PLoS One. 2013; 8: e70809.
